# Improved Monosegment Pedicle Instrumentation for Treatment of Thoracolumbar Incomplete Burst Fractures

**DOI:** 10.1155/2015/357206

**Published:** 2015-05-04

**Authors:** Liehua Liu, Yibo Gan, Qiang Zhou, Haoming Wang, Fei Dai, Fei Luo, Tianyong Hou, Chengmin Zhang, Chen Zhao, Jinsong Zhang, Jianzhong Xu, Yingwen Lü

**Affiliations:** ^1^Department of Orthopedics, Southwest Hospital, Third Military Medical University, Chongqing 400038, China; ^2^Department of Orthopedics, No. 13 People's Hospital of Chongqing, Chongqing 400053, China

## Abstract

*Aim*. Comparing the clinical results of improved monosegment pedicle instrumentation (iMSPI) and short-segment pedicle instrumentation (SSPI) retrospectively. *Method*. 63 patients with thoracolumbar incomplete burst fracture were managed with iMSPI or SSPI. 30 patients were managed with iMSPI and fusion. 33 patients were managed with SSPI and fusion. Operative time, blood loss, postoperative drainage, and complications were recorded. Percentage of anterior body height compression (ABHC%) and sagittal index (SI) were obtained preoperatively, one week postoperatively, and at the last followup. *Results*. The blood loss and postoperative drainage were significantly less in the iMSPI group than in SSPI group (*P* < 0.05). The follow-up duration of the two groups was not significantly different (*P* > 0.05). At 12 months postoperatively posterolateral fusion was obtained satisfactorily. Neither preoperative ABHC% and SI nor postoperative SI were significantly different (*P* > 0.05), but there was a significant difference in postoperative ABHC% (*P* = 0.000). The ABHC% and SI were not significantly different between the two groups at the last followup (*P* > 0.05). There were no fixation failures or other complications. *Summary*. IMSPI yielded satisfactory results similar to those of SSPI in patients with type A3.1/3.2 thoracolumbar fractures. IMSPI is recommended for minor trauma, reducing one-segment fusion, and maximization of the remaining motor function.

## 1. Introduction

Thoracolumbar burst fractures are a common injury in spine surgery. Currently, there are many advocates of surgical treatment for nonstable fractures [1]. In 1986, pedicle screws were first used for thoracolumbar fractures in short-segment fixation and fusion therapy by Roy-Camille et al. [2]. With improvement in the design of pedicle screws and progress in pedicle screw fixation technique, short-segment pedicle instrumentation (SSPI) has achieved satisfactory curative results in patients of thoracolumbar fractures [3, 4]. However, fixation and fusion should be conducted in the levels cephalad and caudad to the fractured vertebral with SSPI, leading to loss of a normal functional spinal unit.

With vertebral fractures mainly occurring in the upper endplate, as long as appropriate methods are used to fix the fractured vertebral and the vertebral cephalad to the fracture, firm fixation and stability can be achieved in some types of fractures as well. The only difference is a reduction in the loss of a motor unit, with better remaining motor function. The single-level fixation was used by Gotzen et al. [5] and Finkelstein et al. [6] for the treatment of some thoracolumbar fractures which causes minor damage to the stability of fractured level only, and they achieved satisfactory results. In recent years, monosegmental pedicle instrumentation (MSPI) has been used for the treatment of thoracolumbar burst fractures and also achieved satisfactory results [7, 8].

The reported MSPI has been used for fixation and/or fusion on a fractured vertebral and the vertebral cephalad to the fracture with monoaxial pedicle screw. As with majority of injuries on upper vertebral, completely screwing into normal bone is very difficult, making it hard to ensure the stability of fixation. In recent years, we have also performed MSPI for the treatment of thoracolumbar incomplete burst fractures, but with improved pedicle screw fixation technology, that is, implanting polyaxial pedicle screws in the fractured vertebral body, while implanting monoaxial pedicle screws in the normal level adjacent to the fractured vertebral. This technique might be a good method to solve the problem of insufficient fixation strength in vertebral.

In this paper, we report a retrospective study of comparing curative effect of improved monosegmental pedicle instrumentation (iMSPI) with SSPI as a control group, for the treatment of thoracolumbar incomplete burst fractures, with more than 1 year of followup.

## 2. Materials and Methods

### 2.1. Clinical Data

From March 2005 to September 2010, 69 cases of thoracolumbar incomplete burst fractures without neurologic deficit were managed with iMSPI or SSPI, with 63 cases followed up and 6 cases dropped out. Basic information from two groups is shown in Table 1.

### 2.2. Inclusion Criteria

The inclusion criteria are (1) thoracolumbar vertebral incomplete burst fracture (AO-ASIF type A3.1 or A3.2); (2) load-sharing score [9] 4–7; (3) both of the pedicles of the injured vertebrae being intact through CT three-dimensional reconstruction; and (4) an age of 18–60 years with nonpathological fractures.

### 2.3. Exclusion Criteria

Exclusion criteria are as follows: (1) patients combined with other major organ damage, such as traumatic brain injury, vital organs injury of the chest or abdomen, and fractures of pelvis, calcaneus, or forearm; (2) multisegmental spinal fractures; (3) osteoporosis (BMD *T* score < −2.5 SD).

### 2.4. Indications for Surgery

(1) In principle, sagittal index (SI) exceeds 15° or loss of anterior body height exceeds 30%; (2) patients could not bear the pain in bed while strongly urged for the surgery.

### 2.5. Surgical Method

**Figure 1:**
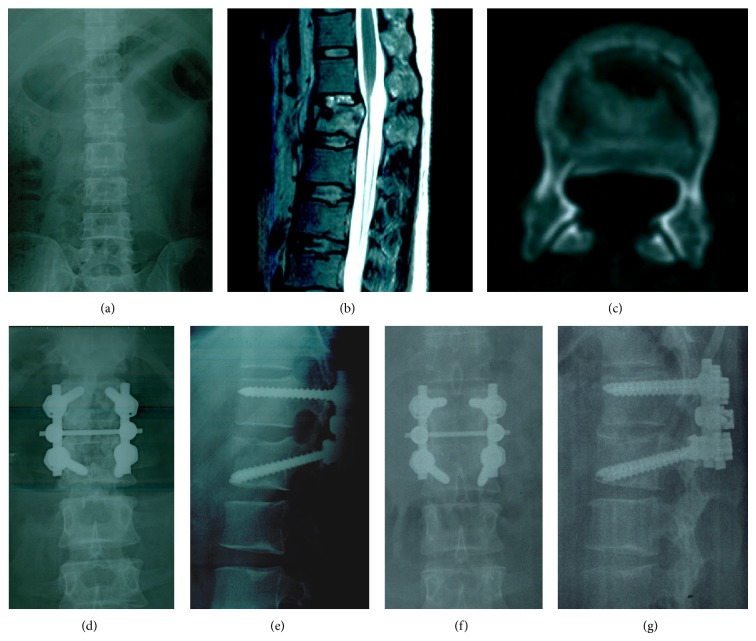
(a) Preoperative anterior-posterior X-ray; (b) preoperative MRI sagittal; (c) preoperative CT cross section; (d) and (e) postoperative radiographs; swing the tail of polyaxial pedicle screws caudally to coincide with the normal curve of spine; (f) and (g) radiographs after 61 months.

All patients were managed with posterior pedicle screw fixation by the same surgeon and his team. Under controlled general anesthesia with endotracheal intubation, the patient was placed in the prone position on a radiolucent operating table with padding for the chest and pelvis area to suspend abdomen. With C-arm X-ray image intensifier, operating table was adjusted to make waist hyperextensive moderately, and a posterior midline incision was made. The first group of 30 patients were managed with iMSPI and fusion, with adequate exposure of plates and transverse process roots of the fractured vertebral and the level cephalad to the fractured vertebral. The pedicle screws were implanted in the levels of fractured vertebral and cephalad to the fractured vertebral. The screws implanted in the upper vertebral were parallel to the cranial endplate using a monoaxial pedicle screw [10]. The screws implanted in the fractured vertebral were towards the front lower end using polyaxial pedicle screws. A spinous process was pressed and manual reduction was conducted. Titanium rods were placed and the tail of polyaxial pedicle screws swings caudally to coincide with the normal curve of spine. Tighten the connection of the polyaxial pedicle screws and titanium rods and prop up alternately to restore vertebral height and to ease the tension on the spinal canal during the bones reduction in virtue of tension of the posterior longitudinal ligament. Lock the monoaxial pedicle screws after fractures reduction was confirmed by X-ray fluoroscopy. The surgeon struck off cartilage surface of facet joint on the fixed segment. After preparation of the graft bed on intervertebral joints, vertebral laminae, and root of transverse processes, fusion was performed with granular allograft. A cross-connector was added between the rods to augment the torsional stability of the construct (Figure 1).

The second group included 33 patients who were managed with six-nail short-segment pedicle screw fixation (SSPI) and fusion, exposing the range of the levels cephalad and caudad to the fractured vertebral. The screw implanting technique was the same as in the first group. Prop up two intervertebral spaces repeatedly for reduction and other operations were the same as the iMSPI group. The primary products which were received by all patients for internal fixation were Moss-Miami (iMSPI group: 19 cases and SSPI group: 20 cases) or Polynices (iMSPI group: 11 patients and SSPI group: 13 patients). The screw size was the same in both groups (45–50 mm in length and 6.0–7.0 mm in diameter). According to intraoperative assessment, the surgeon decided whether to make drainage into incision. In the iMSPI group 15 cases received drainage and in the SSPI group 14 cases received drainage.

### 2.6. Postoperative Treatment

Patients were encouraged to stand and walk 3–5 days postoperatively. Drainage was removed within 48 hours. Thoracolumbar brace was worn 8–12 weeks after operation. X-rays were obtained at postoperative 1 week and 1, 3, 6, and 12 months postoperatively and once a year thereafter.

### 2.7. Observation Items and Methods

Operative time, blood loss, postoperative drainage, and complications were recorded. Visual analogue scale (VAS) was assessed [11] one week postoperatively and at the last followup. Radiographic evaluations were performed by X-ray preoperatively, one week postoperatively, and at the last followup. Twelve months postoperatively, radiograph was observed if the posterolateral fusion was healed by Christensen's standard [12]: “fusion” indicated this quality of fusion at all intended levels, “doubtful fusion” indicated suboptimal quality at intended levels including fusion mass hidden behind the instrumentation, and “nonunion” indicated definite lack of fusion at one of the intended levels. The percentage of anterior body height compression (ABHC%) [8] and sagittal index (SI) [13] were measured. ABHC% was measured as *R* = ([*R*_2_ − *R*_1_]/*R*_2_) × 100% (*R*_1_ is the height of the leading edge of the fractured vertebral and *R*_2_ is the average height of the leading edge of vertebral cephalad and caudad to the fractured one, with *R*_2_ as the reference value of normal vertebral). SI was measured as SI = Cobb angle of kyphosis − angle of normal vertebral; Cobb angle of kyphosis was the angle between lower endplates of injury vertebral and upper normal vertebral. The angle of normal vertebral of T12 and L1 is 0°, while that of L2 was −10°. Internal fixation failure was referred to as a regional convex angle at the last followup exceeding 10° compared to one week postoperatively [14].

### 2.8. Statistical Analysis

The results between groups were compared using the unpaired *t*-test, with the level of significance set at *P* < 0.05, using SPSS version 19.

## 3. Results

Main results in the two groups are shown in Table 2. The amount of blood loss and postoperative drainage were significantly less in the iMSPI group than in SSPI group (*P* < 0.05). At 12 months postoperatively posterolateral fusion was obtained satisfactorily: in iMSPI group, 27 cases and 3 cases achieving “fusion” and “doubtful fusion,” respectively; in SSPI group, 30 cases and 3 cases achieving “fusion” and “doubtful fusion,” respectively, no “nonunion” being observed. There was no significant difference between the two groups in postoperative VAS (*P* = 0.680) and at the last followup (*P* = 0.824).

**Figure 2:**
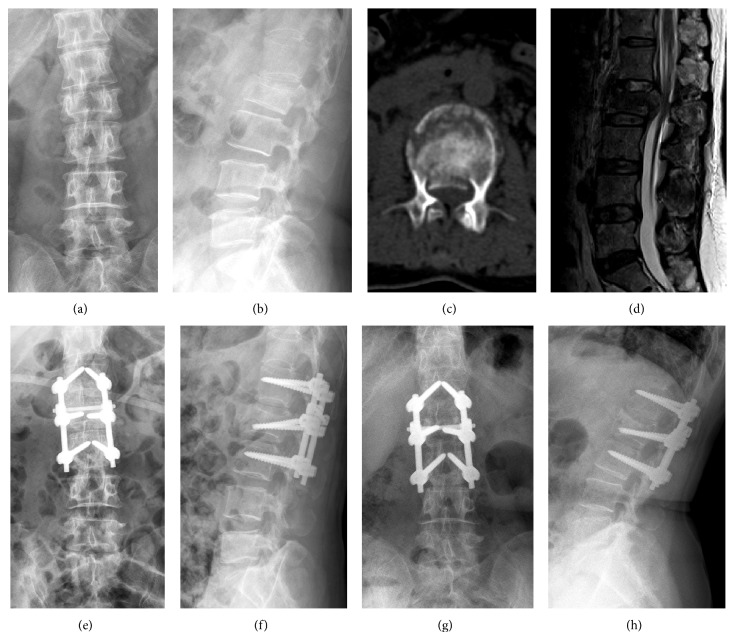
(a) and (b) Preoperative X-ray; (c) preoperative CT cross section; (d) preoperative MRI sagittal; (e) and (f) postoperative radiographs; (g) and (h) radiographs after 24 months.

The followup duration was 48.2 ± 15.9 months (range: 24–77 months) and 52.8 ± 24.7 months (range: 18–109 months) in the iMSPI group and SSPI group, respectively, which was not significantly different (*P* = 0.386). Preoperative ABHC% and SI were not significantly different between the two groups (*P* > 0.05). One week postoperatively, SI was not significantly different between the two groups (*P* = 0.676), but ABHC% was significantly different between the groups (*P* = 0.000), with the SSPI group (2.5% ± 4.2%) having a better vertebral body height restoration than the iMSPI group (8.1% ± 6.0%). At the last followup, ABHC% of iMSPI group was 9.7% ± 6.2%, while for the SSPI group, it was 7.0% ± 5.5%, and there was no significant difference (*P* = 0.072). At the last followup, the SI was 8.2° ± 4.2° in the iMSPI group and 7.6° ± 4.5° in the SSPI group; there was no significant difference (*P* = 0.632). At the last followup, the two groups had no loosening of fixation, displacement or fixation failure, nerve damage, or other complications (Figure 2).

## 4. Discussion

In 1976, Harrington applied his own instruments for the successful treatment of vertebral fracture and dislocation, but wide surgical exposure was necessary, resulting in trauma, excessive bleeding, poor antitorsion of devices, and postoperative complications, such as loosening of internal fixation. In 1985, Dick et al. invented the pedicle screw [15]. Pedicle screw fixation can provide three-dimensional stability and rigid internal fixation [16–18], thus achieving the minimum range of fixation and fusion in injured segment, resulting in widespread clinical application. Currently, SSPI is most commonly used for surgical treatment of thoracolumbar fractures. In the early years, the screws were implanted in the levels cephalad and caudad to the injured vertebral, and the injured vertebral was not screwed, while two motion segments were fixed. However, this procedure had high rates of internal fixation failure and loss of kyphosis correction [14, 19]. In recent years, some studies have reported that implanting screws in the injured vertebral on the basis of four-screw fixation. Comparing with four screws to fix two vertebral bodies, biomechanical research proved that six screws to fix three vertebral bodies decrease the suspension and quadrilateral effects of internal fixation, leading to reducing the stress on the screws and increasing the axial load carrying, antibuckling, and antirotation capability [20, 21]. In addition, other researches demonstrated that vertebral pedicle screws did not make fracture reduction difficult, but the maximum possible distraction of intervertebral space to restore vertebral height and increase tension on the posterior longitudinal ligament to make fracture fragments in the spinal canal achieves reduction. Therefore, in recent years, SSPI has been widely accepted [22].

However, in SSPI, two intervertebral fusions lead to loss of a normal motion segment and impacting the biomechanics of the lumbar spine. The more the fixation and fusion were conducted, the higher the degenerative rate of adjacent segments was [23]. With the passage of time, there would be more problems in older patients. Therefore, surgeons explored whether fixing only one segment to achieve similar curative effects compared to SSPI would prevent the loss of a normal segment. Subsequently, in 1993, McLain et al. [14] reported one-level internal fixation for thoracolumbar wedge compression fractures, and since then many surgeons have attempted to obtain better curative results with MSPI. Therefore, treatment of thoracolumbar burst fractures with MSPI is feasible [8]. But monoaxial pedicle screws are commonly used in MSPI technique making it difficult to ensure that the front end of screws is fixed to normal bone. We have adopted polyaxial pedicle screws for injured vertebral, with the screws fixed in the area of the normal bone as much as possible to obtain better fixation strength. The curative effects have been satisfactory.

In this study, the basic data of the two groups were similar. There were no significant differences in operative time or follow-up duration, but bleeding and postoperative drainage were significantly less in the iMSPI group than in SSPI group. This finding demonstrates that iMSPI has the advantages of less trauma. The SI between the groups was not significantly different preoperatively, postoperatively, or at the last followup. This result indicates that the iMSPI and SSPI groups obtained similar reduction and kyphosis correction, as well as fixation stability. It is noteworthy that postoperative ABHC% is lower in the SSPI group than in the iMSPI group, indicating that height of injured vertebral with six-screw fixation could be recovered satisfactorily. We believe that distraction of double intervertebral space six-screw fixation intraoperatively, compared to distraction of single intervertebral disc, can obtain better restoration of vertebral height. However, at the last followup, there was no significant difference between two groups, because screw-bone interface strength decreased after surgery, because the microfractures appear before complete fusion of posterior bone graft, resulting in decreases in tensile stress of posterior column and increases in anterior column compressive stress, which causes incomplete loss of height of the vertebral body. After having been reduced to a certain degree of vertebral height and bone healing of posterior bone graft, the height of the vertebral body can be maintained and will no longer decrease. At the last followup, the ABHC%, SI, and VASs between the two groups were not significantly different, with no fixation failure. IMSPI retained a normal segment's motor function, with less trauma, lower cost, and so forth, but the indications are narrower than for SSPI, and its reduction was less effective than with SSPI.

Both Wei et al. [8] and Li et al. [24] have reported that MSPI was applied for treatment of thoracolumbar burst fractures and achieved satisfactory curative effect. But Wei et al. reported that there were three cases with loss of correction greater than 10°, including one case of screw loosening at a mean followup of 27.8 months. It is easy to rule out implanting the screw into a normal vertebral bone area incompletely resulting in screw migration and loss of correction. Defino and Scarparo [7] also reported that MSPI resulted in satisfactory radiographic evaluation, clinical evaluation, and functional assessment, but rate of the loss of kyphosis at the last followup was 37.5%. Wei et al. and Li et al.'s rates of kyphosis loss at final followup were 30.2% and 27.8%, respectively. In our study, it was 21.1%. The results showed that iMSPI resulted in better kyphosis correction and better fixation stability. We thought stability of internal fixation was enhanced by long pedicle screws implanted in the area of undamaged bone of the injured vertebrae and cross-connectors fixation.

We believe that the key to successful iMSPI is masterful surgical skills. (1) It is more difficult and complex to conduct reduction with iMSPI than with SSPI. Surgeons must take full advantage of the combination of positioning, techniques, and devices of internal fixation to achieve reduction. (2) IMSPI technology uses polyaxial screws implanting in the fractured vertebral towards the front lower end to maximize fixation strength. By swinging tail of the polyaxial pedicle screw, it is easy to place titanium rods and obtain intervertebral space distraction. Placing cross-connectors was emphasized in order to increase fixation strength. (3) Permanent stability of the damaged segment of the spine requires bone healing of the posterior bone graft. Thus, we advocate striking off surface cartilage of the intervertebral joints, with removing cortical processing of intervertebral joints, combined with posterior bone grafting on vertebral plate for good results of interbody fusion. Based on techniques of screwing, reduction, and other techniques of surgical fixation and fusion in the MSPI we report undergoing some improvements with traditional MSPI; we call it “improved MSPI”.

IMSPI yielded satisfactory therapeutic effect in reduction, fixation, and the effects of the surgery similar to those of SSPI in patients with type A3.1/3.2 thoracolumbar fractures. Moreover, iMSPI causes little trauma and lower cost; iMSPI is recommended for reducing one-segment fixation and fusion and remaining motor function below the level of the injury. Of course, this treatment for thoracolumbar incomplete burst fractures requires longer-term follow-up observation. In clinical practice, proper selection of a surgical approach for thoracolumbar incomplete burst fractures will require addition evidences on the basis of evidence-based medicine for guidance.

## Figures and Tables

**Table 1:** Patients' basic information.

	iMSPI	SSPI	Total	*P* value
Number of cases	30	33	63	
Sex, male : female	17 : 13	19 : 14	36 : 27	0.572^†^
Age (yrs)	42.6 ± 9.6	43.7 ± 9.9	43.1 ± 9.7	0.657
Causes of injury				
Falls	17 (56.7%)	17 (51.5%)	34 (54.0%)	
Traffic accidents	5 (16.7%)	8 (24.2%)	13 (20.6%)	
Crashes	6 (20.0%)	3 (9.1%)	9 (27.3%)	
Heavy parts' fall	2 (6.7%)	5 (15.2%)	7 (11.1%)	
Injured segments (*n*, %)				
T12	4 (13.3%)	8 (24.2%)	12 (19.0%)	
L1	22 (73.3%)	16 (48.5%)	38 (60.3%)	
L2	4 (13.3%)	9 (27.3%)	13 (20.6%)	
AO-ASIF type				
A3.1	14 (46.7%)	14 (42.4%)	28 (44.4%)	
A3.2	16 (53.3%)	19 (57.6%)	35 (55.6%)	
Load-sharing score	5.1 ± 1.1	5.5 ± 0.8	5.3 ± 1.0	0.069
Subject bed time (days)	6.4 ± 3.0	6.4 ± 3.9	6.4 ± 3.5	0.997

^†^Two-tailed Fisher's exact test.

**Table 2:** Main results in the two groups.

	iMSPI group	SSPI group	*P* value
Bleeding (mL)	213 ± 103	324 ± 192	0.006
Operation time (mins)	159 ± 37	174 ± 41	0.135
Drainage (mL)	78 ± 52	142 ± 68	0.008
Posterolateral fusion			
Fusion	27 (90.0%)	30 (90.9%)	0.617^†^
Doubtful fusion	3 (10.0%)	3 (9.1%)	
Nonunion	0 (0.0%)	0 (0.0%)	
SI (°)			
Preoperative	15.3 ± 6.0	18.7 ± 8.4	0.068
One week postoperatively	6.3 ± 4.1	6.7 ± 5.0	0.676
Last followup	8.2 ± 4.2	7.6 ± 4.5	0.632
ABHC (%)			
Preoperative	33.9 ± 11.2	37.8 ± 14.9	0.242
One week postoperatively	8.1 ± 6.0	2.5 ± 4.2	0.000
Last followup	9.7 ± 6.2	7.0 ± 5.5	0.072
Visual analogue scale (VAS)			
One week postoperatively	3.6 ± 0.9	3.8 ± 0.8	0.680
Last followup	0.5 ± 0.8	0.6 ± 0.8	0.824
Follow-up time (months)	48.2 ± 15.9	52.8 ± 24.7	0.386

^†^Chi-square test.
